# Less Is Better: Single-Digit Brain Functional Connections Predict T2DM and T2DM-Induced Cognitive Impairment

**DOI:** 10.3389/fnins.2020.588684

**Published:** 2021-01-11

**Authors:** Haotian Qian, Dongxue Qin, Shouliang Qi, Yueyang Teng, Chen Li, Yudong Yao, Jianlin Wu

**Affiliations:** ^1^College of Medicine and Biological Information Engineering, Northeastern University, Shenyang, China; ^2^Department of Radiology, The Second Affiliated Hospital of Dalian Medical University, Dalian, China; ^3^Key Laboratory of Intelligent Computing in Medical Image, Ministry of Education, Northeastern University, Shenyang, China; ^4^Department of Electrical and Computer Engineering, Stevens Institute of Technology, Hoboken, NJ, United States; ^5^Department of Radiology, Affiliated Zhongshan Hospital of Dalian University, Dalian, China

**Keywords:** resting state fMRI, type 2 diabetes mellitus, cognitive impairment, functional connectivity, machine learning

## Abstract

Type 2 diabetes mellitus (T2DM) leads to a higher risk of brain damage and adversely affects cognition. The underlying neural mechanism of T2DM-induced cognitive impairment (T2DM-CI) remains unclear. This study proposes to identify a small number of dysfunctional brain connections as imaging biomarkers, distinguishing between T2DM-CI, T2DM with normal cognition (T2DM-NC), and healthy controls (HC). We have recruited 22 T2DM-CI patients, 31 T2DM-NC patients, and 39 HCs. The structural Magnetic Resonance Imaging (MRI) and resting state fMRI images are acquired, and neuropsychological tests are carried out. Amplitude of low frequency fluctuations (ALFF) is analyzed to identify impaired brain regions implicated with T2DM and T2DM-CI. The functional network is built and all connections connected to impaired brain regions are selected. Subsequently, L_1_-norm regularized sparse canonical correlation analysis and sparse logistic regression are used to identify discriminative connections and Support Vector Machine is trained to realize three two-category classifications. It is found that single-digit dysfunctional connections predict T2DM and T2DM-CI. For T2DM-CI versus HC, T2DM-NC versus HC, and T2DM-CI versus T2DM-NC, the number of connections is 6, 7, and 5 and the area under curve (AUC) can reach 0.912, 0.901, and 0.861, respectively. The dysfunctional connection is mainly related to Default Model Network (DMN) and long-distance links. The strength of identified connections is significantly different among groups and correlated with cognitive assessment score (*p* < 0.05). Via ALFF analysis and further feature selection algorithms, a small number of dysfunctional brain connections can be identified to predict T2DM and T2DM-CI. These connections might be the imaging biomarkers of T2DM-CI and targets of intervention.

## Introduction

Diabetes mellitus is a common metabolic disorder characterized by hyperglycemia (McCrimmon, et al., 2012). Currently, there are an estimated 463 million adults with diabetes worldwide, of which Type 2 diabetes mellitus (T2DM) accounts for more than 90% (International Diabetes Federation, 2019). The chronic hyperglycemia of T2DM patients may cause systemic damage to nerves, eyes, kidneys, and blood vessels, which may bring many complications, such as cognitive impairment (CI), microvascular complications (Valencia and Florez, 2017),and olfactory dysfunction (Yazla et al., 2018).

T2DM-induced cognitive impairment (T2DM-CI), also known as diabetic encephalopathy, mainly manifests through learning, judgment, and memory deficits, a decline in executive function, and decreased information processing speed (Mijnhout et al., 2006; McCrimmon, et al., 2012; Biessels and Despa, 2018). Many longitudinal studies have found that T2DM is an independent risk factor for Alzheimer’s disease (AD) (Vagelatos and Eslick, 2013) and vascular dementia (VD) (Biessels et al., 2008), and some patients may even deteriorate to severe dementia (Cukierman et al., 2005). However, due to the diversity of clinical manifestations of T2DM-CI and its relatively slow onset, there is no gold standard for diagnosis, which is likely to cause misdiagnosis or missed diagnosis and delay the treatment of patients (Srikanth et al., 2020).

Resting state functional MRI (rs-fMRI) and the subsequent computational analysis have presented the potential of precisely characterizing and inferring neurological diseases, including T2DM-CI (Cohen et al., 2017; Rosenberg et al., 2019). Measures of brain regions and connections are two main aspects of the computational analysis. Amplitude of low frequency fluctuations (ALFF) can reflect the intensity of spontaneous neural activity of each voxel from an energy perspective, thereby reflecting the regularity and physiological state of neuron autonomous activity in different brain regions (Pan et al., 2017). It has been demonstrated that T2DM shows the decreased ALFF in frontal lobe, parietal lobe, and posterior cerebellar lobe (Xia et al., 2013; Cui et al., 2014). ALFF disturbances in the occipital lobe may play an important role in T2DM-related cognitive dysfunction (Wang et al., 2014). Most previous studies have only compared T2DM patients with healthy controls (HC), however, T2DM-CI is not well-studied.

Through various brain atlases [e.g., the recently established human Brainnetome Atlas of 246 brain subregions (Fan et al., 2016)], a whole brain functional network can be constructed from rs-fMRI data to study the brain connections. This method can fully utilize the rich information from the viewpoint of connectomics, find potential neuroimaging biomarkers, and help people understand the neural mechanism of neurological and psychiatric disorders (Craddock et al., 2013; Fornito et al., 2015; Qi et al., 2015; Bassett and Sporns, 2017). Previous studies have shown that T2DM is of aberrant brain functional connectivity (Musen et al., 2012; Chen et al., 2014).

Through machine learning, the integrated models of characteristics across multiple brain connections and regions can be constructed to predict clinical statuses and outcomes (Iniesta et al., 2016; Woo et al., 2017; Dwyer et al., 2018). Remarkable progress has been made for autism, schizophrenia, depression, and AD (Yahata et al., 2016; Sui et al., 2018; Zhu et al., 2019; Jin et al., 2020). Specifically, Liu et al. (2019) selected 23 connections to identify 38 T2DM-CI from 84 T2DM patients and the resulted area under the receiver operating characteristic (ROC) curve (AUC) reached 0.9737.

Better predictive biomarkers of T2DM-CI rest on the effective identification of the discriminative features (or connections). Meanwhile, the number of identified connections must be small to avoid the over-fitting problem in which the fitting errors are artificially smaller than inherent data variance (Whelan and Garavan, 2014; Woo et al., 2017). The resulted model with over-fitting inevitably presents catastrophic generalizability for external data. According to a rule of thumb, 10 samples (patients) are usually required for each feature (or connection) in a binary classifier (Gillies et al., 2016).

Therefore, we propose one effective method of identifying a small number of dysfunctional brain connections and use them as imaging biomarkers to distinguish among T2DM-CI, T2DM with normal cognition (T2DM-NC), and healthy controls (HC). There are three contributary aspects. First, one ALFF-based way is proposed to identify dysfunctional connections through the impaired Brainnetome regions, integrating the information of both brain regions and connections. Second, 6, 7, and 5 dysfunctional connections have been identified as biomarkers distinguishing between T2DM-CI and HC, T2DM-NC and HC, and T2DM-CI and T2DM-NC. The strength of identified connections are significantly different among groups and correlated with cognitive assessment score (*p* < 0.05). Third, the constructed three models can predict T2DM and T2DM-CI with the AUC higher than 0.90. These identified dysfunctional brain connections might direct the underlying neural mechanism of T2DM-CI and the potential targets of intervention of T2DM care. The ALLF-based method can be expanded to study other neurological disorders.

## Materials and Methods

### Participants

A total of 53 T2DM patients who met the diagnostic criteria were recruited from Affiliated Zhongshan Hospital of Dalian University from January 2015 to January 2017. Inclusion criteria for T2DM patients were that they must: (1) meet the diagnostic criteria for diabetes, (2) be 45 to 75 years old, (3) have a history of diagnosis of 5 to 10 years, and (4) be right-handed. Meanwhile 39 healthy people who were examined at Affiliated Zhongshan Hospital of Dalian University at the same time were recruited as the HC group. The sex, age, and education level of the HC group were matched with T2DM patients. Exclusion criteria for all participants were: (1) patients with vision, hearing, language communication, or physical activity difficulties; (2) patients with psychiatric disorders or head trauma; (3) alcoholics, smoking addicts, or drug abusers; (4) MRI contraindications; and (5) patients with brain injury, cerebral hemorrhage, cerebral infarction, and other brain diseases, and patients with brain white matter demyelination (Age-Related White Matter Changes (ARWMC) score >1). The detailed demographic information of the enrolled subjects is shown in Table 1. This study was approved by the ethics committee of Affiliated Zhongshan Hospital of Dalian University and was in accordance with the 1964 Helsinki declaration and its later amendments or comparable ethical standards. All the subjects were informed about the examination, expressed their knowledge of the study, and signed their informed consent.

**TABLE 1:** Demographic, clinical, and neuropsychological information of the participants.

Characteristics	T2DM-CI	T2DM-NC	HC	*p*-value
Gender (male/female)	12/10	18/13	23/16	0.138
Age	62.64 ± 4.94	59.56 ± 7.56	58.34 ± 6.69	0.092
BMI (kg/m^2^)	25.99 ± 3.03	25.78 ± 3.16	25.10 ± 2.49	0.458
Education duration	10.23 ± 2.89	11.35 ± 3.09	10.86 ± 2.73	0.387
T2DM duration	10.14 ± 4.66	9.15 ± 6.74	−	0.406
FPG (mmol/L)	14.13 ± 7.31	11.08 ± 7.93	5.24 ± 0.29	0.013*
MoCA	21.91 ± 2.77	27.16 ± 1.15	27.24 ± 1.15	< 0.001*
CDT	2.45 ± 0.59	2.77 ± 0.61	3.03 ± 0.57	0.003*
VFT	20.55 ± 6.44	23.63 ± 5.92	22.85 ± 6.42	0.205
AVLT	21.38 ± 5.69	24.94 ± 4.64	25.65 ± 3.33	0.003*
DST	10.59 ± 1.89	12.19 ± 2.62	12.09 ± 2.12	0.025*
TMT(s)	69.95 ± 27.57	51.77 ± 26.83	51.90 ± 21.14	0.019*

Data are presented as mean ± SD. BMI, body mass index; FPG, fasting plasma glucose; MoCA, Montreal Cognitive Assessment; CDT, clock drawing test; VFT, verbal fluency test; AVLT, auditory verbal learning test; DST, digit span test; TMT, trail making test. * indicates a difference of p < 0.05, which is statistically significant.

We used neuropsychological tests, including the Chinese version of the Montreal Cognitive Assessment (MoCA) (Nasreddine et al., 2005), clock drawing test (CDT) (Shulman, 2000), auditory verbal learning test (AVLT) (Powell et al., 1991), digit span test (DST) (Melikyan et al., 2019), trail making test (TMT) (Llinàs-Reglà et al., 2017), and verbal fluency test (VFT) (Weiss et al., 2003), to determine the cognitive status of T2DM patients. The same trained physicians judged whether T2DM patients have cognitive impairment and divided them into T2DM-CI (MoCA score < 26, *n* = 22) and T2DM-NC (MoCA score ≥ 26, *n* = 31). The details are given in Table 1.

### Rs-fMRI Data Acquisition

MRI scanning was performed using one Magnetom 3.0 Tesla scanner (Siemens, Germany) with a 12-channel head phased array surface coil. The gradient field is 45 mT/m, and the gradient switching rate is 200 mT/ms. The subject’s head was fixed with a sponge pad before scanning and was informed to keep their head still during the scan. Structural images were acquired using the standard 3D magnetization prepared rapid gradient echo (MPRAGE) sequence: repetition time (TR) = 2530 ms, echo time (TE) = 2.22 ms, slice thickness = 1.0 mm, flip angle (FA) = 7°, field of view (FOV) = 224 × 224 mm, matrix = 224 × 224, layers = 192. Rs-fMRI images were collected by the echo planar imaging (EPI) pulse sequence: TR = 2000 ms, TE = 30 ms, slice thickness = 3.5 mm, FA = 90°, FOV = 224 × 224 mm, matrix = 64 × 64, layers = 31. 240 time phases were collected and 240 images were obtained. The MRI images will be available upon reasonable request after approval by the Ethic Committee of Affiliated Zhongshan Hospital of Dalian University.

### Overview of the Study Procedure

**FIGURE 1:**
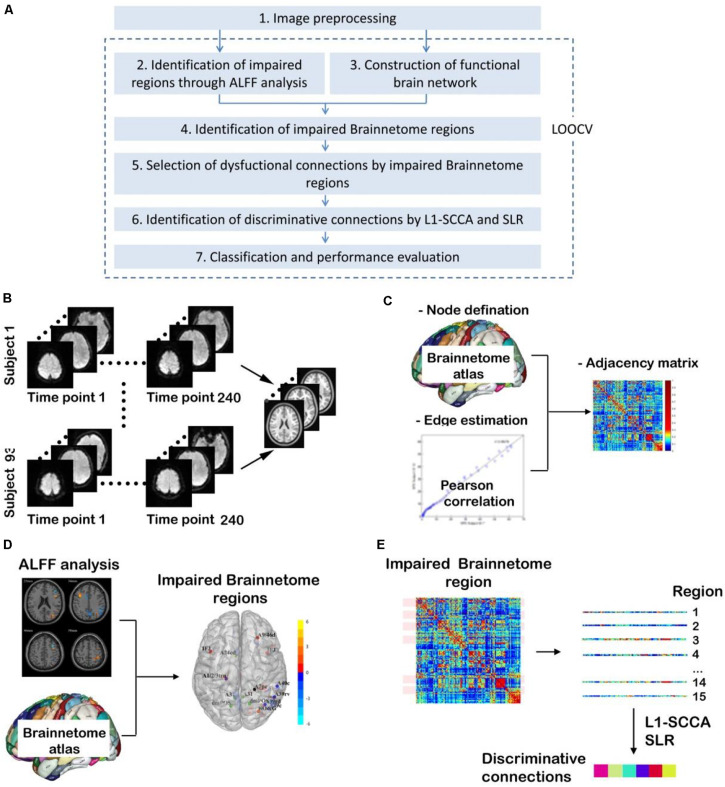
Overview of study procedure. **(A)** Main steps in this study [It is noted that steps from (2) to (7) are carried out many times since they are included in a procedure of leaving-one-out cross validation (LOOCV) to avoid the leakage of category information]; **(B)** Image preprocessing; **(C)** Construction of functional brain network; **(D)** Identification of impaired Brainnetome subregions by ALFF analysis; **(E)** Identification of discriminative connections by L1-SCCA and spare logistic regression (SLR).

As shown in Figure 1, there are seven steps in our study. (1) Image processing is performed according to the standard procedures. (2) ALFF analysis is done to identify the impaired regions for three two-group comparisons. (3) Functional brain network is constructed for each participant. (4) Impaired Brainnetome regions are identified. (5) Dysfunctional connections connected with the impaired Brainnetome are selected. (6) Discriminative connections are identified by L_1_-norm regularized sparse canonical correlation analysis (L_1_-SCCA) and sparse logistic regression (SLR). (7) Classifiers are trained, and their performance is evaluated.

It is noted that, to avoid category information leakage, steps from (2) to (7) in Figure 1 are carried out in a procedure of leaving-one-out cross validation (LOOCV). It means that steps from (2) to (7) have be conducted for n1, n2, and n3 times for T2DM-CI versus HC, T2DM-NC versus HC, and T2DM-CI versus T2DM-NC, where n1, n2, and n3 are the number of participants in three classifications after step (1) of imaging preprocessing.

### Image Preprocessing

In this study, resting state fMRI data are preprocessed using Data Processing and Analysis for Brain Imaging (DPABI) toolkit^1^ in MATLAB 2018b software. As shown in Figure 1B, at first, the initial 10 time points of fMRI data are removed to exclude the influence of the instability of equipment initialization and subjects’ adaptation to the environment. Second, slice-timing correction and realignment for head motion correction are carried out. Three participants with head motion exceeding 2.0 mm maximum translation or 2° rotation are excluded. Third, detrending and nuisance covariates regression, including Friston 24-parameter model, and mean time series of global, white matter, and cerebrospinal fluid signals as regressors, are conducted to remove the influence of physiological factors. Fourth, spatial normalization is carried out, and the brain structure of each subject is normalized to the standard template by the Diffeomorphic Anatomical Registration Through Exponentiated Lie Algebra (DARTEL) tool (Ashburner, 2007). Finally, images are smoothed by Gaussian of full-width at half-maximum 4 mm. Because ALFF analysis is then needed, we have skipped filtering during the preprocessing. It is noted that 89 subjects (19 T2DM-CI, 31 T2DM-NC, and 39 HC) took part in the following study since three subjects were removed during image preprocessing.

### ALFF Calculation and Statistical Analysis

We used the modules in the DPABI toolkit to calculate ALFF. First, time series of each voxel are transformed into frequency domain by Fourier transform and then the power spectrum is obtained. Subsequently, the square root of each frequency power spectrum is calculated according to the frequency band (usually 0.01–0.08 Hz), and the mean value is ALFF. Finally, the ALFF values are standardized to reduce the errors caused by individual differences. The standardized ALFF value is the ALFF value of each voxel divided by the whole brain ALFF mean value.

We have performed statistical analysis on the standardized ALFF values of T2DM-CI, T2DM-NC, and HC. The ALFF values among the three groups are compared by one-way ANOVA test and the statistical map with significant difference is used to create a mask. Then two-sample *t*-tests are performed as *post hoc* tests to identify regions with significant differences in the mask above. The significance level of two-sample *t*-tests are set at *p* < 0.05 with 1000 permutations corrected with the threshold-free cluster enhancement (TFCE) correction. It is found that permutations corrected with the TFCE correction can best balance the family-wise error (FWE) rate and test-retest reliability (Chen et al., 2018).

To avoid the category information leakage, ANOVA test and two-sample *t*-test are carried out after leaving one out, not for all subjects. Specifically, in each fold of the leave-one-out procedure, we have conducted the above ANOVA test and two-sample *t*-test on all subjects except the one who is taken out, then we get the regions with significantly different ALFF of this fold. They are named the impaired regions. In summary, this step of ALFF calculation and statistical analysis has been conducted 58 times for T2DM-CI versus HC, 70 times for T2DM-NC versus HC, and 50 times for T2DM-CI versus T2DM-NC.

### Construction of Functional Brain Networks

The network consists of many nodes and edges between those nodes. In the functional brain network, nodes represent brain regions and edges represent the degree of statistical dependence of blood oxygen level dependent (BOLD) imaging between different brain regions. As shown in Figure 1C, the present study has used the Human Brainnetome Atlas (Fan et al., 2016), which parcellates the whole human brain into 246 subregions, and each subregion represents a node in the brain network. With the progress of MRI scanning, changes in activity of different brain regions can be reflected as time courses. For each subject, we obtain the average time courses of the 246 brain subregions and then we calculate the Pearson correlation coefficient between the average time courses of any two subregions as a functional connectivity indicator between them, which can be used as the edge of the brain network. After that, we can get one 246 × 246 adjacency matrix of each subject, which is called the weighted functional connectivity matrix.

### Identification of Impaired Brainnetome Subregions

After using two-sample *t*-test of ALFF to determine voxels with significant differences between two groups, we excluded clusters with less than 20 voxels. Spatially matching the impaired regions identified by ALFF analysis with Brainnetome Atlas can determine the volume of the impaired region in each Brainnetome subregions. We sort the Brainnetome subregions by volume of impaired region from large to small. Finally, two lists of subregions with increased and decreased ALFF are obtained for each two-group comparison.

Previous studies have reported that, compared with HCs, T2DM patients have decreased ALFF values in brain regions which are related to cognitive impairment. There are 15 Brainnetome subregions with decreased ALFF for both T2DM-CI versus HC and T2DM-NC versus HC, which are named as the impaired Brainnetome subregions. For T2DM-CI versus T2DM-NC, there are only 10 subregions with decreased ALFF. In order to get the same number of subregions for the three groups, we have added five subregions with increased ALFF.

### Identification of Connections With High Discriminative Power

In constructed adjacency matrix, all functional connections connected to the 15 impaired Brainnetome subregions are considered to be potentially discriminative.

The number of features is still too large for classification. We utilize a combination of L_1_-SCCA and SLR to further perform dimension reduction (Yahata et al., 2016). At first, we have two data matrices: the first data matrix of ${\text{X}}_{1}={\left[{\text{x}}_{1}^{1},{\text{x}}_{1}^{2},\ldots ,{\text{x}}_{1}^{N}\right]}^{\text{T}}$ and the second matrix of ${\text{X}}_{2}={\left[{\text{x}}_{2}^{1},{\text{x}}_{2}^{2},\ldots ,{\text{x}}_{2}^{N}\right]}^{\text{T}}$ . X_1_ lists the attributes all subjects with a dimension of *N* × *p*_1_ (*N* is the number of subjects, *p*_1_ is 3 here). The first column of X_1_ is the “Diagnosis” label (either 0 or 1), while the second and third columns are the age and gender (1 for male, 0 for female). X_2_ lists the connections connected with 15 impaired Brainnetome subregions with a dimension of *N* × *p*_2_ (*p*_2_ is 3570 here). L_1_-SCCA is applied to get the sparse projection matrices V_1_ and V_1_ from X_1_ and X_2_. As the equation given in references (Witten et al., 2009; Yahata et al., 2016), for a canonical variable, L_1_-SCCA is formulated as,

(1) $$\begin{array}{c} \max_{{\text{v}}_{1},{\text{v}}_{2}}{\text{v}}_{1}^{\text{T}}{\text{X}}_{1}^{\text{T}}X_{2}{\text{v}}_{2}\;\mathrm{subject}\mathrm{to}{||{\text{v}}_{1}||}_{1}^{2}\leq \lambda_{1},{||{\text{v}}_{2}||}_{1}^{2}\leq \lambda_{2},{||{\text{v}}_{1}||}_{2}^{2}\\ \leq 1,{||{\text{v}}_{2}||}_{2}^{2}\leq 1 \end{array}$$

where v_1_ and v_2_ are the projection vectors and λ_1_ and λ_2_ are their sparseness, respectively. Subsequently, the canonical variable only associated with the “Diagnosis” label is determined, the connections corresponding to the diagnostic canonical variable is chosen, and the effect of nuisance variables of age and gender is reduced.

Sparse logistic regression is further used to reduce the dimension of features and identify the connections with high discriminative power. Given *N* feature-label data samples {(*x*_1_,*y*_1_),…,(*x*_N_,*y*_N_)}, LR aims to find the parameter vector θ such that the likelihood function *l*(θ) is maximized.

(2) $$l\left(\theta \right)=\sum_{n=1}^{N}\left[y_{n}\text{log}p_{n}+\left(1-y_{n}\right)\text{log}\left(1-p_{n}\right)\right]$$

where,

(3) $$p_{n}=\frac{1}{1+\text{exp}\left(-f\left(x_{n};\theta \right)\right)}$$

here $f\left(x_{n};\theta \right)=\sum_{d=1}^{D}\theta_{d}x_{d}+\theta_{0}$, *D* is the dimension of features and θ_0_ is the bias.

Sparse logistic regression combines the automatic relevance determination (ARD) with LR (Yamashita et al., 2008). Imposing the constraint on the weight parameter, ARD assumes that each parameter θ_*d*_ has a Gaussian prior with mean 0.

(4) $$P\left(\theta_{d}|\alpha_{d}\right)=N\left(0,\alpha_{d}^{-1}\right)\;d=1,\ldots ,D$$

here α_*d*_ is the inverse variance of the normal distribution and it is treated as a hyper-parameter, named “the relevance parameter.” α_*d*_ regulates the range of θ_*d*_. It is known that most α_*d*_ diverges to infinity and the corresponding θ_*d*_ is pruned. Finally, the connections related to the label are automatically selected by SLR.

After this reduction, an average of 15.47 connections remain. However, these surviving connections are still too many for the sample in this study and result in over-fitting. Therefore, we instigate the influence of surviving connections on the classification performance and determine the final discriminative connections when the highest performance reaches this point.

### Classification and Performance Evaluation

Support vector machine (SVM) is used to build prediction models. This study has used the library for support vector machines (LIBSVM) toolkit^2^, which integrates functions such as SVM kernel selection, parameter adjustment, and prediction. We chose the radial basis function (RBF) as the kernel function of the SVM, and the values of the optimal penalty coefficient *C* and the kernel function parameter *Gamma* are determined by the grid search method through 5-fold cross validation.

Due to the limited number of samples in this study, we have used LOOCV to estimate the generalization of the classifier. The receiver operating characteristic (ROC) curve, the area under ROC curve (AUC), and the confusion matrix are used to quantify the performance of the classifier. Moreover, using the fixed discriminative connections identified in this study as the features, three SVM models are trained and evaluated by LOOCV.

## Results

### Impaired Brainnetome Subregions Determined by ALFF

**FIGURE 2:**
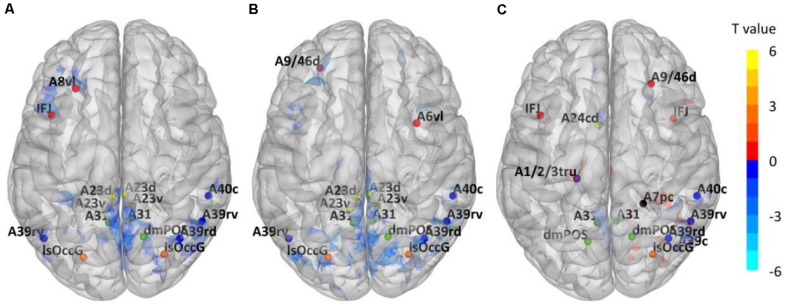
Impaired Brainnetome subregions determined by ALFF. **(A)** T2DM-CI versus HC; **(B)** T2DM-NC versus HC; **(C)** T2DM-CI versus T2DM-NC.

Through ALFF analysis and subsequent matching, 15 impaired subregions have been identified for T2DM-CI versus HC, T2DM-NC versus HC, and T2DM-CI versus T2DM-NC (Figure 2 and Table 2). In Figure 2, the abbreviation of brain subregion is used, and one can refer to the original paper for the full names (Fan et al., 2016).

**TABLE 2:** Impaired brain subregions identified through ALFF analysis in T2DM-CI versus HC, T2DM-NC versus HC, and T2DM-CI versus T2DM-NC.

ID	Brain subregion	Anatomical and cyto-architectonic descriptions	MNI coordinate			Selected or not		
			*X*	*Y*	*Z*	CI vs. HC	NC vs. HC	CI vs. NC
15	MFG_L_7_1	A9/46d, dorsal area 9/46	−27	43	31		**√**	
16	MFG_R_7_1	A9/46d, dorsal area 9/46	30	37	36			**√**
17	MFG_L_7_2	IFJ, inferior frontal junction	−42	13	36	**√**		**√**
18	MFG_R_7_2	IFJ, inferior frontal junction	42	11	39			**√**
23	MFG_L_7_5	A8vl, ventrolateral area 8	−33	23	45	**√**		
26	MFG_R_7_6	A6vl, ventrolateral area 6	34	8	54		**√**	
132	SPL_R_5_4	A7pc, postcentral area 7	23	−43	67			**√**
136	IPL_R_6_1	A39c, caudal area 39 (PGp)	45	−71	20			**√**
138	IPL_R_6_2	A39rd, rostrodorsal area 39 (Hip3)	39	−65	44	**√**	**√**	**√**
142	IPL_R_6_4	A40c, caudal area 40 (PFm)	57	−44	38	**√**	**√**	**√**
143	IPL_L_6_5	A40c, caudal area 40 (PFm)	−47	−65	26	**√**	**√**	
144	IPL_R_6_5	A39rv, rostroventral area 39 (PGa)	53	−54	25	**√**	**√**	**√**
151	Pcun_L_4_3	dmPOS, dorsomedial parietooccipital sulcus	−12	−67	25			**√**
152	Pcun_R_4_3	dmPOS, dorsomedial parietooccipital sulcus	16	−64	25	**√**	**√**	**√**
153	Pcun_L_4_4	A31, area 31 (Lc1)	−6	−55	34	**√**	**√**	**√**
154	Pcun_R_4_4	A31, area 31 (Lc1)	6	−54	35	**√**	**√**	**√**
161	PoG_L_4_4	A1/2/3tru, area 1/2/3 (trunk region)	−21	−35	68			**√**
175	CG_L_7_1	A23d, dorsal area 23	−4	−39	31	**√**	**√**	
176	CG_R_7_1	A23d, dorsal area 23	4	−37	32	**√**	**√**	
181	CG_L_7_4	A23v, ventral area 23	−8	−47	10	**√**	**√**	
182	CG_R_7_4	A23v, ventral area 23	9	−44	11	**√**	**√**	
183	CG_L_7_5	A24cd, caudodorsal area 24	−45	7	37			**√**
209	sOcG_L_2_2	lsOccG, lateral superior occipital gyrus	−22	−77	36	**√**	**√**	
210	sOcG_R_2_2	lsOccG, lateral superior occipital gyrus	29	−75	36	**√**	**√**	**√**

*ID, the ID of the note (brain subregion) in the Human Brainnetome Atlas; MFG, middle frontal gyrus; SPL, superior parietal lobule; IPL, inferior parietal lobule; Pcun, precuneus; PoG, postcentral gyrus; CG, cingulate gyrus; sOcG, superior occipital gyrus. √ indicates the brain region which is selected as the impaired brain subregions.*

Using T2DM-CI versus HC as an example, we have conducted the group comparison (or ALFF analysis) 58 times in LOOCV loop. During these 58 comparisons, only one sample (or patient) is different between any two comparisons. Some clusters of voxels with significantly different ALFF will be obtained and there is only a slight difference (a several of voxels) between the “impaired regions” of any two comparisons. However, this slight difference has been eliminated in the impaired Brainnetome subregions. It is because these Brainnetome subregions are selected if they overlap with the “impaired regions” and the overlap status does not change with the slight variation of “impaired regions.” We have compared the identified subregions in 58 experiments of LOOCV loop for T2DM-CI versus HC and found they are completely the same. It is also true for the other two comparisons.

For T2DM-CI versus HC, two impaired subregions are in the frontal lobe, four in the inferior parietal lobule, three in the precuneus, four in the cingulate gyrus, and two in the occipital lobe. For T2DM-NC versus HC, there is the same spatial distribution as for T2DM-CI versus HC.

Two subregions in the middle frontal gyrus belong to the executive control network (ECN), four in the inferior parietal, four in the cingulate gyrus, and three in the precuneus are in the default model network (DMN). Two in the occipital lobe belong to the visual network (VN). Among 15 subregions, 13 are overlapped between T2DM-CI versus HC and T2DM-NC versus HC, indicating that T2DM-CI and T2DM-NC have a common neuropathological basis.

For T2DM-CI versus T2DM-NC, three subregions are in the frontal lobe, one in the superior parietal lobule, four in the inferior parietal lobule, one in the postcentral gyrus, four in the precuneus, one in the cingulate gyrus, and one in the occipital lobe. Among 15 subregions, seven have appeared in the above two comparisons and they belong to DMN and ECN. One subregion in the superior parietal lobule and one in the postcentral gyrus are the new ones which do not appear in the other comparisons. From the viewpoint of intrinsic brain network, three subregions (ID: 16, 17, 18) are in ECN, nine in DMN (ID: 136, 138, 142, 144, 151, 152, 153, 154, 183), one in FPN (ID: 132), 1 in DAN (ID: 161), and one in VN (ID: 210).

### Dysfunctional Connections With High Discriminative Power

**FIGURE 3:**
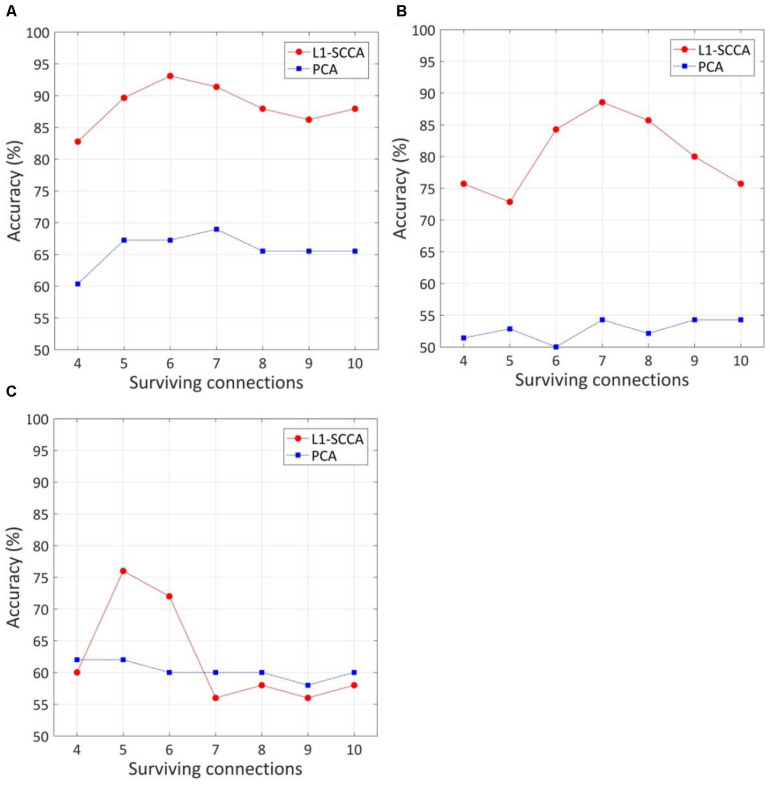
The effect of the number of discriminative connections on prediction accuracy and comparison of our method with feature reduction of principle component analysis (PCA). **(A)** T2DM-CI versus HC; **(B)** T2DM-NC versus HC; **(C)** T2DM-CI versus T2DM-NC.

The effect of the number of discriminative connections on prediction accuracy is given in Figure 3. It is shown that the SVM model has the highest accuracy of 93.1%, while six discriminative connections remain for T2DM-CI versus HC. For T2DM-NC versus HC and T2DM-CI versus T2DM-NC, the optimal number of discriminative connections is seven and five. The feature selection method of L1-SCCA and SLR is much better than the dimension reduction of principal component analysis (PCA).

**FIGURE 4:**
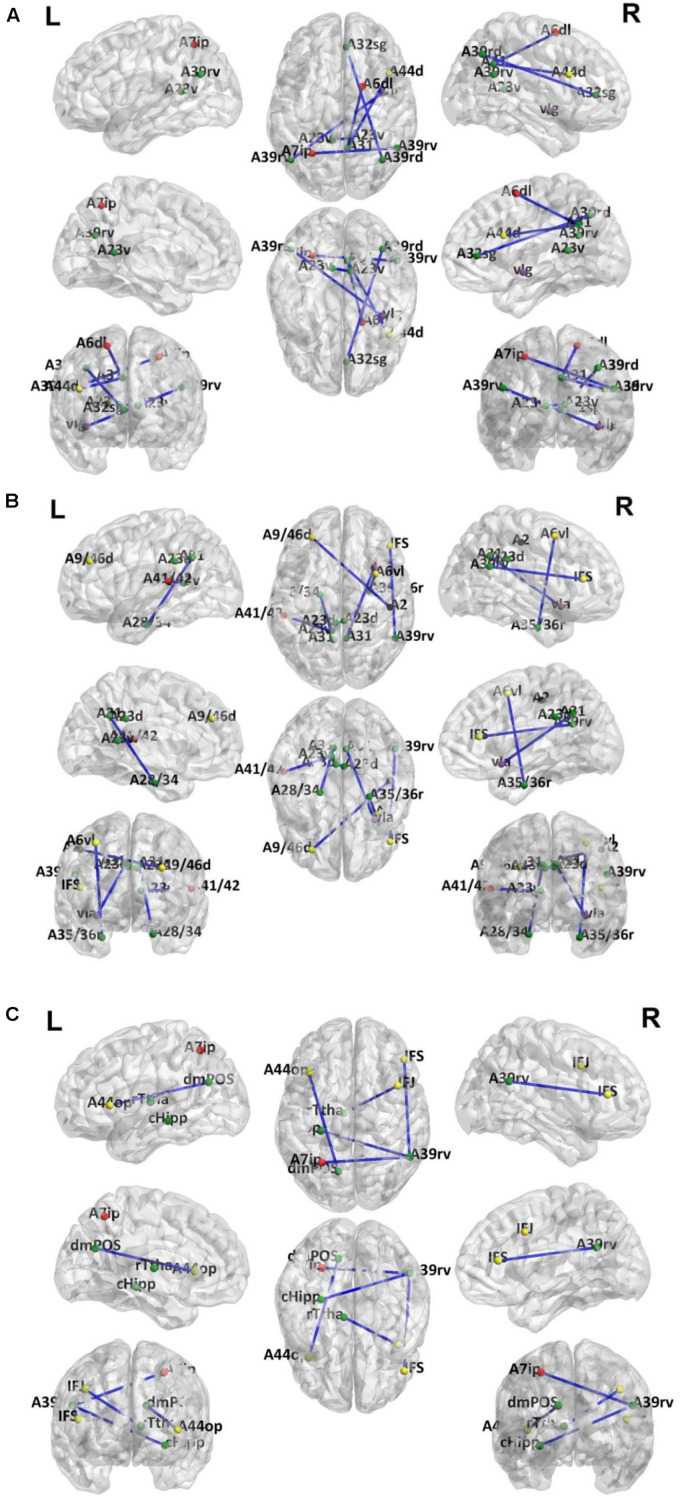
Dysfunctional connections with high discriminative power. **(A)** Six connections for T2DM-CI versus HC; **(B)** Seven connections for T2DM-NC versus HC; **(C)** Five connections for T2DM-CI versus T2DM-NC. The blue lines represent connections used for classification. Dots represent brain subregion nodes connected to these connections. Different colored dots represent subregions from different resting state network. The green dots indicate subregions of DMN, the red dots indicate subregions of FPN, yellow dots indicate subregions of ECN, purple dots indicate subregions of SAN and black dots indicate subregions of DAN.

Because LOOCV is used to divide the dataset, the surviving connections for each fold are slightly different. Sorting the connections by repeat times, the top six, top seven, and top five connections are listed in Tables 3–5 for T2DM-CI versus HC, T2DM-NC versus HC, and T2DM-CI versus T2DM-NC, respectively. The spatial locations of the identified connections are given in Figure 4. The straight-line distance between two endpoints of each connection is also calculated according to the MNI coordinates of the subregions and presented in Tables 3–5.

**TABLE 3:** Six discriminative functional connections for T2DM-CI versus HC.

Connected nodes ID	Connected brain subregions	Repeat times	Distance (mm)	Resting state network	Correlation with MoCA	*p*-value
181-182 (A23v-A23v)	CG_L_7_4, CG_R_7_4	58	17.29	DMN	0.3247	0.0129
133-144 (A7ip-A39rv)	SPL_L_5_5, IPL_R_6_5	58	85.24	FPN-DMN	–0.3465	0.0077
8-154 (A6dl-A31)	SFG_R_7_4, Pcun_R_4_4	57	69.86	FPN-DMN	–0.4403	0.0005
138-188 (A39rd-A32sg)	IPL_R_6_2, CG_R_7_7	56	117.63	DMN	–0.3506	0.0070
30-154 (A44d-A31)	IFG_R_6_1, Pcun_R_4_4	55	80.75	ECN-DMN	–0.3482	0.0074
143-170 (A39rv-vId/vIg)	IPL_L_6_5, INS_R_6_4	37	112.21	DMN-SAN	–0.3746	0.0038

*CG, cingulate gyrus; SPL, superior parietal lobule; IPL, inferior parietal lobule; SFG, superior frontal gyrus; Pcun, precuneus; IFG, inferior frontal gyrus; INS, caudodorsal posterior insula; DMN, default mode network; FPN, frontoparietal network; ECN, executive control network; SAN, salience network.*

**TABLE 4:** Seven discriminative functional connections for T2DM-NC versus HC.

Connected nodes ID	Connected brain subregions	Repeat times	Distance (mm)	Resting state network	Correlation with MoCA	*p*-value
154-166 (A31-vIa)	Pcun_R_4_4, INS_R_6_2	70	87.50	DMN-SAN	–0.0712	0.5581
26-110 (A6vl-A35/36r)	MFG_R_7_6, PhG_R_6_1	70	88.66	ECN-DMN	–0.0858	0.4800
71-181 (A41/42-A23v)	STG_L_6_2, CG_L_7_4	70	48.43	FPN-DMN	0.0316	0.7950
175-176 (A23d-A23d)	CG_L_7_1, CG_R_7_1	70	8.31	DMN	0.0081	0.9472
32-144 (IFS-A39rv)	IFG_R_6_2, IPL_R_6_5	70	89.94	ECN-DMN	0.0108	0.9296
15-160 (A9/46d-A2)	MFG_L_7_1, PoG_R_4_3	66	102.00	ECN-DAN	0.0354	0.7709
115-153 (A28/34-A31)	PhG_L_6_4, Pcun_L_4_4	64	78.19	DMN	–0.1040	0.3915

*Pcun, precuneus; INS, caudodorsal posterior insula; MFG, middle frontal gyrus; PhG, parahippocampal gyrus; STG, superior temporal gyrus; CG, cingulate gyrus; IFG, inferior frontal gyrus; IPL, inferior parietal lobule; PoG, postcentral gyrus; DMN, default mode network; SAN, salience network; ECN, executive control network; FPN, frontoparietal network.*

**TABLE 5:** Five discriminative functional connections for T2DM-CI versus T2DM-NC.

Connected nodes ID	Connected brain subregions	Repeat times	Distance (mm)	Resting state network	Correlation with MoCA	*p*-value
32-144 (IFS-A39rv)	IFG_R_6_2, IPL_R_6_5	50	89.94	ECN-DMN	–0.4273	0.0020
18-237 (IFJ-rTtha)	MFG_R_7_2, Tha_L_8_4	50	63.64	ECN-DMN	0.3831	0.0060
37-151 (A44op-dmPOS)	IFG_L_6_5, Pcun_L_4_3	50	96.28	ECN-DMN	0.3862	0.0056
144-217 (A39rv-cHipp)	IPL_R_6_5, Hipp_L_2_2	48	91.44	DMN	0.3555	0.0113
133-144 (A7ip-A39rv)	SPL_L_5_5, IPL_R_6_5	37	85.24	FPN-DMN	–0.3527	0.0120

*IFG, inferior frontal gyrus; IPL, inferior parietal lobule; MFG, middle frontal gyrus; Tha, Thalamus; Pcun, precuneus; Hipp, hippocampus; SPL, superior parietal lobule; IPL, inferior parietal lobule; ECN, executive control network; DMN, default mode network; FPN, frontoparietal network.*

It is found that for T2DM-CI versus HC, among the six selected connections (Table 3, Figure 4A), two are between regions within DMN (left and right subregions in cingulate gyrus; two subregions in cingulate gyrus and inferior frontal gyrus), two between DMN and frontoparietal network (FPN), one between DMN and ECN, and one between DMN and salience network (SAN). DMN appears in all six connections. All six connections are long-distance links across different lobes; three of the six are inter-hemispheric, and the other three are right intra-hemispheric. Though the straight-line distance between subregions of 181 and 182 is only 19.29 mm, it has been treated as a “long-distance” link as it is inter-hemispheric. No left intra-hemispheric connection is observed. For the three inter-hemispheric connections, one subregion in the precuneus (ID: 154, Pcun_R_4_4) appears twice.

For T2DM-NC versus HC, among the seven selected connections (Table 4 and Figure 4B), there were two between regions of DMN, two between DMN and ECN, one between ECN and dorsal attention network (DAN), one between DMN and FPN, and one between DMN and SAN. Among the seven connections, two are left intra-hemispheric, two are inter-hemispheric, and three are right intra-hemispheric. All seven connections are long-distance links. The connection between subregions of 175 and 176 is inter-hemispheric, though the straight-line distance is only 8.31 mm.

When comparing T2DM-CI versus HC and T2DM-NC versus HC, it is surprising to find that no overlap exists between the six and seven connections although DMN, FPN, ECN, and SAN are involved in both cases. It indicates that the neuropathological substrate for T2DM-CI and T2DM-NC might be different from the viewpoint of functional connections, though they have almost the same impaired subregions (Table 2). This finding may emphasize that the information of brain regions and connections are intrinsically different and complementary.

For T2DM-CI versus T2DM-NC, among the five selected connections (Table 5, Figure 4C), there are three between DMN and ECN, one between subregions within the DMN, and one between DMN and FPN. Three connections are with the subregion of IPL_R_6_5 in the inferior parietals lobule. Three are inter-hemispheric connections. All five connections are long-distance links across different lobes, suggesting that the global integration of information, not the local communication, might be abnormal in T2DM-induced cognitive impairment. The hippocampus and thalamus are new regions which do not appear in T2DM-CI versus HC and T2DM-NC versus HC.

### Altered Strength of Discriminative Connections

**FIGURE 5:**
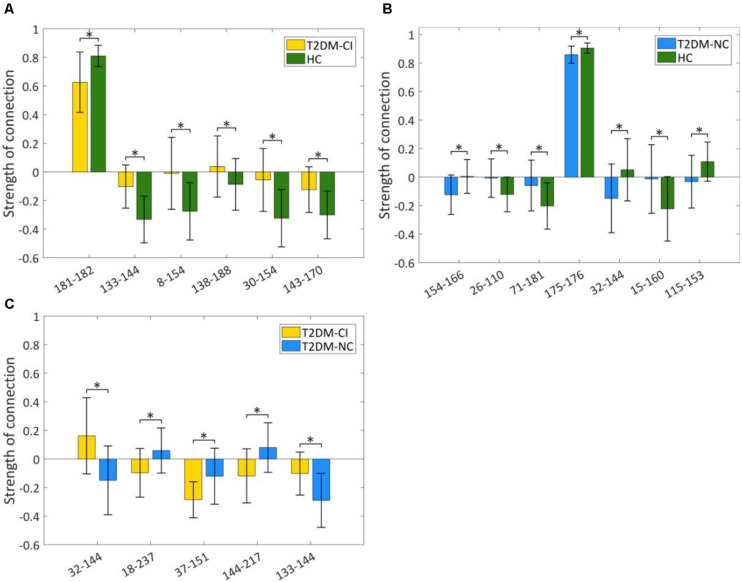
Strength comparison of dysfunctional discriminative connections. **(A)** Six connections for T2DM-CI versus HC; **(B)** Seven connections for T2DM-NC versus HC; **(C)** Five connections for T2DM-CI versus T2DM-NC. * indicates a difference of *p* < 0.05, which is statistically significant.

The strength of discriminative connections is compared between different groups (Figure 5). All discriminative connections have significantly different strengths (*p* < 0.05). Here we define that the smaller connectivity indicates a more negative strength of connection and the greater connectivity indicates a more positive. As shown in Figure 5A, T2DM-CI shows the smaller connectivity in one connection (181-182) but the greater connectivity in five connections than HC. Most discriminative connections are “weak.” Specifically, only one connection has a strength higher than 0.6 and the other five have strength less than 0.4.

For T2DM-NC versus HC, one connection has strength higher than 0.8 and the other six have strength less than 0.3. Three connections have greater connectivity in T2DM-NC than HC (26-110; 71-181; 15-160) and the other four show the opposite results.

For T2DM-CI versus T2DM-NC, all five discriminative connections are “weak” and with an absolute strength less than 0.3. Three connections in T2DM-CI show smaller connectivity than T2DM-NC (18-237; 37-151; 144-217), but two show the greater connectivity.

### Strength of Discriminative Connections and MoCA Score

The correlations between the real value of five discriminative connections and MoCA score are analyzed and the correlation coefficients (*r*) and *p*-values are listed in Tables 3–5 for three comparisons. For T2DM-CI versus HC, the strength of all six connections is significantly correlated with MoCA score. The first connection (181-182) has positive *r* of 0.3247, corresponding to the one with the smaller connectivity in T2DM-CI, and the others have negative *r*. As expected, there are no significant correlations between the strength of discriminative connections and MoCA score for T2DM-NC versus HC because they have a similar MoCA score >26.

**FIGURE 6:**
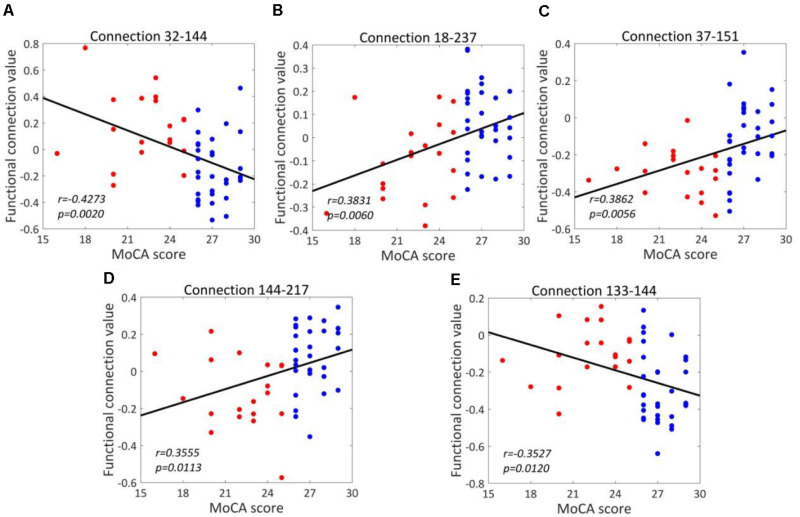
Correlations between the dysfunctional discriminative connections and MoCA scores for T2DM-CI and T2DM-NC groups. **(A)** Connection 32-144; **(B)** Connection 18-237; **(C)** Connection 37-151; **(D)** Connection 144-217; **(E)** Connection 133-144.

For T2DM-CI versus T2DM-NC, the correlations between the real value of five discriminative connections and MoCA score are given in Figure 6. It is found that they are significantly correlated (*p* < 0.05) and the correlation coefficient (*r*) is −0.4273, 0.3831, 0.3862, 0.3555, and −0.3527. For the connection between the inferior frontal gyrus and inferior parietal lobule, the value is positive in T2DM-CI but negative in T2DM-NC (ID: 32 and 144). The same trend occurs for the connection between the superior temporal gyrus and inferior parietal lobule (ID: 133 and 144). The opposite trend appears for the other three connections: middle frontal gyrus and Thalamus (ID: 18 and 237); inferior frontal gyrus and precuneus (ID: 37 and 151); and inferior parietal lobule and hippocampus (ID: 144 and 217).

We have analyzed the correlations between the strength of five discriminative connections in T2DM-CI versus T2DM-NC and CDT, AVLT, DST, TMT, VFT, respectively. The Pearson correlation coefficient (*r*) and the *p*-values are calculated. Only three cases are significant (*p* < 0.05): Connection 32-144 and CDT (*r* = −0.3137); Connection 18-237 and DST (*r* = 0.3191); and Connection 144-217 and CDT (*r* = 0.2894). Since the five discriminative connections are determined according to the classification label given by the MoCA threshold, their strength is significantly correlated with MoCA (Table 5). However, only three of 25 cases are significant for the five neuropsychological test scales of CDT, AVLT, DST, TMT, and VFT. A possible reason might be that these scales measure different aspects of the neuropsychology or cognition of T2DM patients.

### Performance of Predictive Models

**FIGURE 7:**
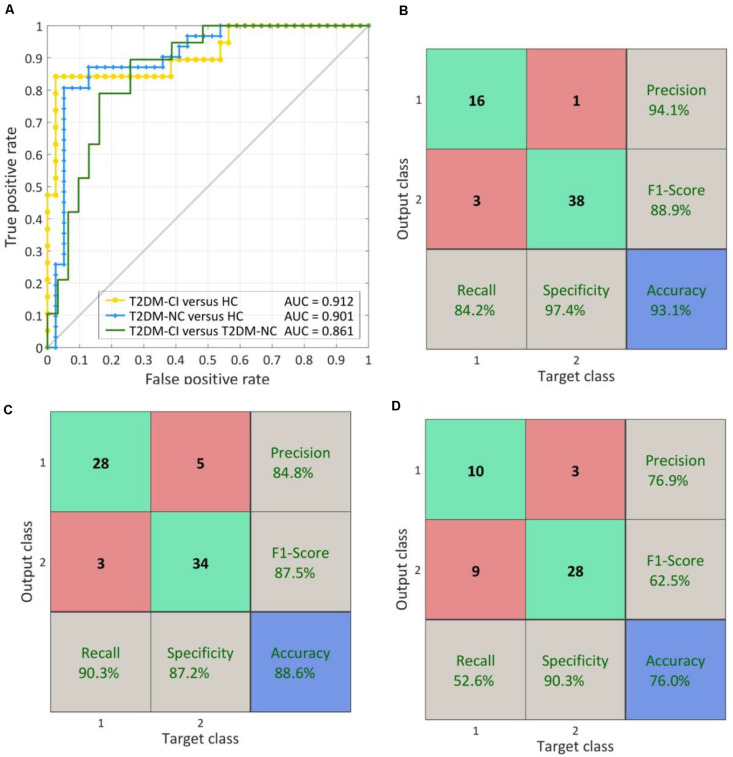
The ROC curve and confusion matrix obtained by LOOCV procedure. **(A)** The ROC curves for three classifications; **(B)** The confusion matrix of T2DM-CI versus HC; **(B)** The confusion matrix of T2DM-NC versus HC; **(D)** The confusion matrix of T2DM-CI versus T2DM-NC.

As shown in Figure 7A, for T2DM-CI versus HC, the optimal SVM models achieve an average accuracy of 93.1% and an AUC of 0.912 in the LOOCV loop. The precision, F1-score, recall, and specificity are 94.1, 88.9, 84.2, and 97.4%, respectively (Figure 7B.

For T2DM-NC versus HC (Figures 7A,C), the optimal SVM models achieve an average accuracy of 88.6% and an AUC of 0.901. The precision, F1-score, recall, and specificity are 84.8, 87.5, 90.3, and 87.2%, respectively. The performance is slightly lower than the models for T2DM-CI versus HC.

For T2DM-CI versus T2DM-NC (Figures 7A,D), the optimal SVM models achieve an average accuracy of 76.0% and an AUC of 0.861. However, the recall and F1-score are lower and only reach 62.5 and 52.6%, respectively. Of the nineteen patients with T2DM-CI, nine are wrongly predicted as T2DM-NC.

**FIGURE 8:**
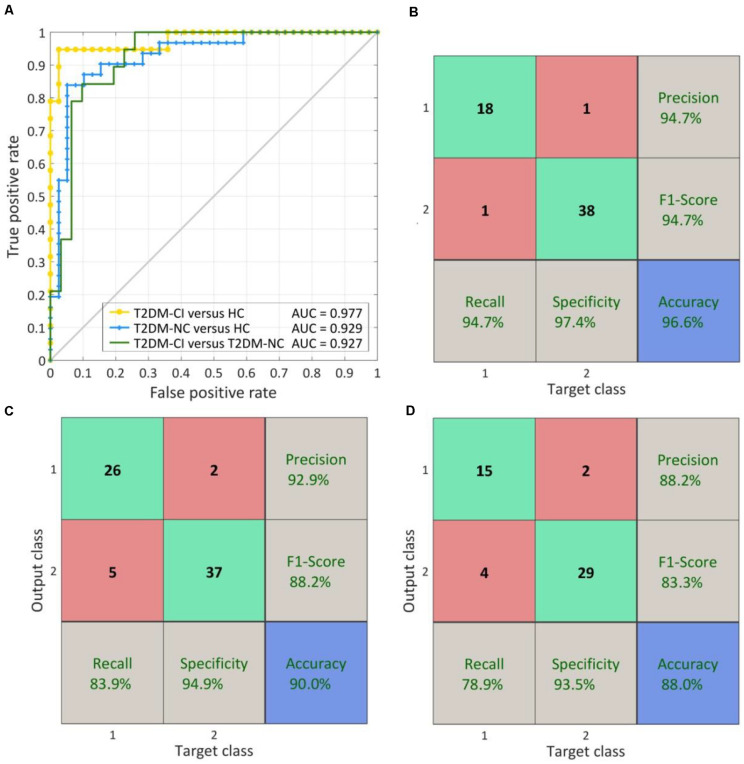
The ROC curve and confusion matrix obtained by fixed discriminative connections. **(A)** The ROC curves for three classifications; **(B)** The confusion matrix of T2DM-CI versus HC; **(C)** The confusion matrix of T2DM-NC versus HC; **(D)** The confusion matrix of T2DM-CI versus T2DM-NC.

When using the fixed discriminative connection as input features, the performance of SVM models can be improved. As shown in Figure 8, the AUC can be increased to 0.977, 0.929, and 0.927 for T2DM-CI versus HC, T2DM-NC versus HC, and T2DM-CI versus T2DM-NC, respectively. Especially for T2DM-CI versus T2DM-NC, the recall and F1-score can reach 78.9 and 83.3%, respectively, although four patients with T2DM-CI are still predicted wrongly.

## Discussion

To the best of our knowledge, this is the first study to identify a small number of dysfunctional brain connections as imaging biomarkers distinguishing among T2DM-CI, T2DM-NC, and HC simultaneously. As small as six, seven, and five identified connections can lead to reliable SVM classifiers and the prediction accuracy can reach 96.6, 90.0, and 88.0% for T2DM-CI (*n* = 19) versus HC (*n* = 39), T2DM-NC (*n* = 31) versus HC (*n* = 39), and T2DM-CI (*n* = 19) versus T2DM-NC (*n* = 31), respectively. The small number of connections alleviates the over-fitting problem. The proposed new way of identifying connections starts from ALFF analysis to find impaired Brainnetome subregions, further selects discriminative connections from ones linked with impaired subregions by L1-SCCA and SLR, and determines the final connections through investigating the effect of the number of connections on prediction accuracy.

### Impaired Brainnetome Subregions for ALFF

Compared with the HC group, the 15 impaired Brainnetome subregions with decreased ALFF in the two T2DM groups (T2DM-CI and T2DM-NC) are mostly the same, located in the frontal lobe, inferior parietal lobule, precuneus, posterior cingulate gyrus, and occipital lobe. This finding is in line with previous studies. The frontal lobe is involved in cognitive functions such as execution function, attention, memory, and language (Chayer and Freedman, 2001); the precuneus is related to many high-level cognitive functions, such as episodic memory, self-related information processing, and self-awareness (Cavanna and Trimble, 2006). The decreased activity in the occipital lobe is significantly correlated with visual memory decline, information processing speed loss, and attention loss. In addition, a relevant study has reported that the hypometabolism and neural degeneration in the posterior cingulate cortex are related to cognitive decline in AD, schizophrenia, and other brain diseases (Dan et al., 2019). Zhou et al. concluded that the inferior parietal lobule, including the angular gyrus and the supramarginal gyrus, is involved in higher cognitive function activities, especially executive control functions (Zhou et al., 2019). The decreased ALFF reflects the inhibition of neurons in related brain regions and the decrease of activity (Wang et al., 2011).

For T2DM-CI versus T2DM-NC, 12 subregions belong to DMN and ECN and the other three belong to FPN, DAN, and VN. These regions appear in AD, mild cognitive impairment, and schizophrenia, and are thought be implicated with cognition (Sui et al., 2018; Jin et al., 2020). In summary, the identified Brainnetome subregions are impaired from the viewpoint of ALFF (i.e., the intensity of spontaneous neural activity) and might help understand the neuropathological basis of T2DM and T2DM-CI.

### Discriminative Connections Are DMN-Related and Long-Distance

For three classifications, the identified brain connections with high discriminative power are mainly between subregions within DMN and between DMN and other resting state networks including ECN, FPN, and SAN. It is no wonder that DMN are implicated with T2DM and T2DM-CI (Yang et al., 2016; Macphersona et al., 2017). DMN is related to continuous thinking, imagination, and internal mental activities such as memory, theory of mind, and self-thinking (Brewer et al., 2011). In addition, DMN is considered to be related to human cognitive function (Broyd et al., 2009), and some studies have also found that abnormal activity in the DMN is closely related to some psychiatric disorders, such as MCI (Wang et al., 2019), AD (Agosta et al., 2012) and schizophrenia (Jing et al., 2019).

In T2DM-CI versus T2DM-NC, it is found that most of the discriminative connections are between DMN and other resting state networks. ECN is involved in goal-oriented advanced cognitive tasks and plays an important role in adaptive cognitive control (Seeley et al., 2007). FPN is related to interoceptive awareness, working memory, and emotional regulation (Salas et al., 2014), and studies have found that the destruction of FPN and DMN is the basis of metacognitive deficits (Jia et al., 2020). Combining the functions of these networks, previous research, and the findings found in this study, we speculated that the cognitive impairment caused by T2DM may be mainly related to the abnormal connectivity patterns between DMN and ECN, FPN, or other resting state networks.

Another finding is that all discriminative connections for three classifications are long-distance. It is in agreement with the report of T2DM-CI (Liu et al., 2019). One possible reason is that the impaired subregions are hub nodes in the brain network and they mediate the long-distance connections between brain modules (Crossley et al., 2014). The hubs are generally implicated in different brain disorders. These long-distance connections are functionally valuable for information integration and are closely related with cognition (van den Heuvel et al., 2012; Crossley et al., 2013).

### The Methodology From Brain Regions to Connections

Here we have proposed one way of identifying discriminative connection for the diagnosis prediction of T2DM and T2DM-CI. It belongs to the category of “From brain regions to connections” and the measure of brain regions is ALFF. Our previous study used prior knowledge to localize the etiological origin of depression (lateral habenula, LHb), selected discriminate connections linked with LHb, and realized an accurate prediction of subclinical depression (Zhu et al., 2019). This method is also in the category of “From brain regions to connections.” Moreover, the measure of brain regions can be certainly expanded to other fMRI measures, including regional homogeneity (ReHo) and Voxel-mirrored Homotopic Connectivity (VMHC).

The identified Brainnetome subregions help narrow the search range of discriminative connections. More importantly, the impaired Brainnetome subregions will leave “ALFF memory” to the discriminative connections so that the final classification has used valuable information of both brain region and connections. We observed that among 15 impaired subregions, 13 are overlapped between T2DM-CI versus HC and T2DM-NC versus HC. However, no overlap exists between the six and seven discriminative connections. This observation suggests that the information of brain regions and connections are intrinsically different and complementary.

Another category of identifying discriminative connections is “Select connections from network directly.” The selection method can be L1-SCCA, SLR, elastic net, and so on (Yahata et al., 2016; Liu et al., 2019). These methods emphasize the role of connections and believe the hypothesis of “the node is determined by its connections.” The third category is “Select brain regions and connections simultaneously.” The measures of brain regions and connections are treated equally, and the selection or reduction of measures rely on powerful machine leaning algorithms or multiple variable analysis (Jin et al., 2020).

### Less Is Better for Reliable Biomarkers

Over-fitting is one of the main issues for neuroimaging-based classifiers of neurological disorders. We have 30135 connection candidates (246^∗^245/2 = 30135), and more if necessary. However, the sample size is only 92 in this study due to the difficulty of recruiting patients. An effective way of identifying discriminative features (or connections) is the key to generating better predictive biomarkers.

Less is better for the reliable biomarkers. In this study, single-digit brain functional connections have been identified and enable prediction of T2DM and T2DM-IC. Specifically, six, seven, and five dysfunctional connections can distinguish between T2DM-CI and HC, T2DM-NC and HC, and T2DM-CI and T2DM-NC, respectively. Each feature (or connection) corresponds to 10 samples (patients) in a binary classifier (Gillies et al., 2016). Fewer connections can alleviate the problem of over-fitting and increase the generalizability of prediction models. Fewer connections means that the etiological origin of T2DM and T2DM-CI is more specific and potential intervention will be targeted and precise.

It should be noted that our study aims to identify a small number of dysfunctional brain connections as imaging biomarkers distinguishing between T2DM-CI, T2DM-NC, and HC. These identified dysfunctional brain connections may help to understand the underlying neural mechanism of T2DM-CI and even find targets of intervention. However, for real clinical diagnosis and intervention, more studies are required. For clinical diagnosis of T2DM-CI, a reasonable way might be to conduct the cognitive assessment from the clinic at first to find the high-risk group and then to do an fMRI scan.

### Limitations and Future Directions

There are many limitations in the current study. The sample size is still small, although the total number has reached 92. Moreover, the generalizability of the classifier is not tested on an independent validation cohort since all participants are recruited from one single center. However, the results of this study have confirmed the potential of functional connectivity patterns based on ALFF results to predict cognitive impairment in T2DM patients. In the future, more effective prediction models may be obtained through larger sample data combined with data from different sources.

In terms of the construction of the prediction model, for the time being, only the combination of L_1_-SCCA and sparse logistic regression are used to reduce the dimension of selected features. In the future, we can use elastic net model, minimum-redundancy maximum relevancy, recursive feature elimination, and other feature selection and dimension reduction methods to obtain a better classification model (Liu et al., 2019).

In this study, T2DM patients have been divided into T2DM-CI and T2DM-NC according to neuropsychological tests. However, because T2DM patients may suffer from diabetic microangiopathy, diabetic retinopathy, and other complications, these diseases may also affect ALFF and functional connectivity. In future research, it may be necessary to consider the impact of other T2DM complications and analyze the potential impact of factors such as the course of T2DM patients and the degree of cognitive impairment (Rosenberg et al., 2019).

Finally, this study mainly analyzed from the perspective of brain functional connection network through fMRI data. In the future research, we can combine more neuroimaging data to find abnormalities caused by T2DM-induced cognitive impairment from structural abnormalities as a comprehensive biomarker, so as to make a more reliable analysis and diagnosis of the disease (Woo et al., 2017; Jin et al., 2020).

## Conclusion

In this study, via ALFF analysis and effective algorithms of feature selection, single-digit dysfunctional brain connections have been identified to predict T2DM and T2DM-induced CI. Only using six, seven, and five discriminative connections, the trained SVM models can realize the classification between T2DM-CI and HC, T2DM-NC and HC, and T2DM-CI and T2DM-NC, with an AUC of 0.912, 0.901, and 0.861, respectively. The strength of identified connections were significantly different among groups and correlated with cognitive assessment (MoCA) score. The impaired Connectome subregions and dysfunctional connections might serve as the imaging biomarkers of T2DM-CI and as potential targets of intervention of T2DM care. The developed method leaves “ALFF memory” to the discriminative connections so that the final classification has used valuable information from both brain regions and connections, which can be expanded to studies of other neurological disorders.

## Data Availability Statement

The MRI images will be available upon reasonable request after approval by the Ethic Committee of Affiliated Zhongshan Hospital of Dalian University.

## Ethics Statement

The studies involving human participants were reviewed and approved by the Ethics Committee of Affiliated Zhongshan Hospital of Dalian University. The patients/participants provided their written informed consent to participate in this study.

## Author Contributions

SQ, YY, and JW designed and directed the study. HQ, DQ, YT, and CL analyzed the data. DQ and JW recruited participants and acquired the data. HQ, SQ, YT, and YY drafted the manuscript together. All authors revised and approved the final version of the manuscript.

## Conflict of Interest

The authors declare that the research was conducted in the absence of any commercial or financial relationships that could be construed as a potential conflict of interest.

## Footnotes

1 http://www.restfmri.net

2 https://www.csie.ntu.edu.tw/~cjlin/libsvm/
